# Personalized Integrated Care Promoting Quality of Life for Older People: Protocol for a Multicenter Randomized Controlled Trial

**DOI:** 10.2196/47916

**Published:** 2023-07-24

**Authors:** Elda Judica, Peppino Tropea, Raquel Bouça-Machado, Mayca Marín, Elisa Calarota, Liviu Cozma, Raluca Badea, Mona Ahmed, Michael Brach, Joaquim J Ferreira, Massimo Corbo

**Affiliations:** 1 Department of Neurorehabilitation Sciences Casa di Cura Igea Milan Italy; 2 Campus Neurológico Sénior Torres Vedras Portugal; 3 Asociación Parkinson Madrid Madrid Spain; 4 Wohlfahrtswerk für Baden-Wurttemburg Stuttgart Germany; 5 University of Medicine and Pharmacy Bucharest Romania; 6 University and Emergency Hospital of Bucharest Bucharest Romania; 7 Institute of Sport and Exercise Sciences Münster University Münster Germany

**Keywords:** Parkinson disease, dementia, neurodegenerative, chronic diseases, health care technologies, integrated care

## Abstract

**Background:**

Alzheimer disease (AD) and Parkinson disease (PD) are the 2 most common neurodegenerative diseases affecting millions of people worldwide. The *Personalized Integrated Care Promoting Quality of Life for Older People* (PC4L) project proposes an integrated, scalable, and interactive care ecosystem that can be easily adapted to the needs of several neurodegenerative and chronic diseases, care institutions, and end user requirements.

**Objective:**

The study protocol developed within the framework of the PC4L project aims to iteratively test the integrated platform and its modules, and focuses primarily on assessing the impact of the proposed solution (ie, the PC4L platform) on patients’ quality of life, as well as its usability and feasibility on a large-scale sample size in 3 different scenarios (home, neurorehabilitation, and day care centers).

**Methods:**

A prospective multicenter clinical study is conducted in 5 European countries (Germany, Italy, Portugal, Romania, and Spain) at 6 different pilot centers, for 3 months, in patients with PD, Parkinsonism, AD, and other dementias (ODs). Patients were randomized in a ratio of 1:1 to the intervention group (use of the PC4L system) or the control group (no intervention). The PC4L system consists mainly of a wristband for monitoring parameters such as steps and levels of physical activity, and the PC4L app, which includes different engaging functionalities. Both groups are assessed through baseline and end-of-study clinical evaluations, including assessment of quality of life through the EQ-5D-3L scale.

**Results:**

The study protocol is part of a project approved and funded by the European Commission Horizon 2020 (grant agreement number 875221). The ethics committees of all involved centers reviewed and approved the study protocol. The study began with the recruitment phase in September 2022, and enrollment ended in February 2023. Recruitment is now closed (April 2023). The results of this study are expected to be published in summer 2023. A total of 558 patients, 279 per study group, were recruited. The results will allow to clarify the impact of PC4L on quality of life, will assess the empowerment of patients and the medical resources use, as well as the usability of the final version of the PC4L system. It will also provide information on the support of the system as a tool to facilitate the decision-making process.

**Conclusions:**

The PC4L project intends to test a technology-based, integrated, scalable, and interactive care platform on patients with neurodegenerative diseases and proposes a good coordinated care model between all involved actors. Future developments of the PC4L solution may involve caregivers and socio-health professionals in the decision-making process in order to facilitate efficient communication between all stakeholders and ensure reliable and protected access to data within Europe.

**Trial Registration:**

ClinicalTrials.gov NCT05538455; https://clinicaltrials.gov/study/NCT05538455

**International Registered Report Identifier (IRRID):**

DERR1-10.2196/47916

## Introduction

### Background

There is an urgent need to increase the efficiency and sustainability of health and social care systems across Europe, as there is a growing trend in public spending, which is expected to reach 14% of gross domestic product by 2030 [1]. The aging of the population, accompanied by an increase in chronic diseases, including cardiovascular diseases, diabetes, asthma, mental and physical disorders, and neurodegenerative conditions, represents the main cause of this situation [2,3]. In fact, the existence of comorbidities and the confluence of several chronic diseases are progressively more frequent in older adults, which increases the need to develop models and tools to improve integrated health care systems.

The aging of the population has also led to major reforms in long-term care policy and systems in many European Union (EU) countries, raising the necessity for alternatives. This implies the need for help with household tasks or other practical errands, transport to hospital/clinic visits or social visits, social companionship, emotional guidance, or organizing professional care. In most European countries, much of the care for people older than 60 years is based on informal care [4].

Among the most common chronic diseases in older adults, neurocognitive disorders, such as dementia, Alzheimer disease (AD), and Parkinson disease (PD), are the most disabling. Today, more than 10 million Europeans live with neurocognitive impairment disorders at different stages of dementia.

In recent years in Europe, digitalization is recognized as a powerful tool to achieve many potential benefits such as improving quality of life and putting patients at the center of the care process [5].

Therefore, the current situation can be improved by the creation of an integrated care platform, capable of establishing correlations between comorbidities, investigating the use of polypharmacy, mitigating potential health risks, studying the social variables involved, and promoting unified treatment procedures or social services. This solution could help patients, caregivers, and social health professionals to better monitor several diseases, while also considering the social context [6-8]. Besides, according to a recent work [9], the inclusion of a web platform for monitoring signs and symptoms of older persons by health care professionals over time has proved to be an important resource.

In addition, people with chronic diseases experience difficulties in their daily lives and require specialized care services as well as treatments, imposing high burdens on the public budget: this requires special attention to adequately address the sustainability of the social health system in Europe.

Personalized Integrated Care Promoting Quality of Life for Older People (PC4L) is a European Commission Horizon 2020 project (grant agreement number 875221) that proposes an integrated, scalable, and interactive care ecosystem, and can be easily adapted to the reality of several chronic diseases, care institutions, and end user requirements, benefiting all the involved actors, from patients to caregivers and health professionals. Its main contributions consist of the following:

- Building an integrated, scalable, and interactive care ecosystem for neurodegenerative diseases and adaptable to other chronic conditions
- Finding the best actions and measures from a medical and social point of view that can facilitate an improved quality of life, awareness, and care management for senior users with neurodegenerative and other chronic diseases
- Providing a personalized recommendation and interaction model, which can support the user through gamification techniques, with the final goal of adopting healthy habits, maintaining a good daily routine, and following the prescribed actions by the professionals
- Enabling multidisciplinary communication between all involved stakeholders, facilitating better time management for social and health professionals, and contributing to a cost-efficient, flexible, and highly adaptable solution for senior users with short- or long-term conditions

By adopting a user-centered design approach starting from the early stages of the project [10], the inclusion of the needs and requirements of the end users has been crucial toward the PC4L platform development and implementation [11] and the current outline of the protocol presented here. In fact, through the work performed during the first part of the project, we identified the more important factors from the personal, social, and health points of view that are able to influence the daily lives of the end users and reflect on their expectations regarding the PC4L solution.

### Aims

This study represents the third, and last, phase (pilot 3) of large-scale pilots aiming to gradually co-design and iteratively test the PC4L integrated platform and modules.

While pilot phases 1 and 2 focused on technical feasibility and patients’ satisfaction, this protocol focuses on the following objectives:

- To assess the impact of using the PC4L platform on patients’ quality of life
- To compare the metrics generated by the PC4L platform in real-life conditions with the clinical outcome measures
- To evaluate the feasibility and safety of measuring disease progression and patient health status through the global functioning of the PC4L system
- To assess and compare the feasibility and usability of 2 different versions of the PC4L system (fully equipped version vs cloud-based solution)
- To increase patients’ safety and empowerment

As the primary outcome, the study will provide:

- The difference in quality of life between groups (experimental vs control group), measured by the EQ-5D-3L scale at the end of the study

The following secondary outcomes will also be acquired:

- The correlation coefficient of the PC4L platform metrics (eg, steps and physical activity levels, sleep, number of falls, cognitive scores) with the clinical outcome measures (eg, motor clinical evaluation, sleep questionnaire, Falls Efficacy Scale-International [FES-I], cognitive evaluations) measured at baseline, end of the study, and the values of change from baseline
- System usability assessment, measured through the System Usability Scale (SUS), at the end of the study
- Users’ satisfaction with the PC4L system, measured through a 5-point Likert Scale, the mHealth User Satisfaction Questionnaire, and the participants’ answers to open questions, at the end of the study
- Changes in patients’ satisfaction and empowerment, measured by the Patient Assessment of Chronic Illness Care (PACIC) and the Short Assessment of Patient Satisfaction (SAPS) scales, respectively, at baseline and at the end of the study
- Change in patients’ use of medical resources (number of admissions and number of days spent in health institutions) in the last month, measured at baseline and at the end of the study

## Methods

### PC4L Platform

#### Instrumental Setup

The PC4L ecosystem consists of the following:

- Wearable sensors that are used by the patient for monitoring disease-related parameters in passive and interactive paradigms (eg, simple biomeasurements such as heart rate or complex biomeasurements such as motor behavior; passive measurement if initiated automatically, and interactive measurement if initiated by the patient). The wearable sensor used is the smartwatch Fitbit Versa 2 (Google LLC). Patients will wear the sensor in the dominant arm
- The PC4L app for processing data directly from the smartphone (Samsung A20 or another similar model) inertial measurement unit sensors and GPS and directly from users. In particular, the data collected will be anthropometric data and symptoms, as well as data related to medication uptake, which are to be provided directly by the patient or the caregiver, via short questionnaires and annotations. Data related to motor behaviors through GPS and inertial sensors
- Depth camera that will be deployed in the clinical centers (rehabilitation) for monitoring motor parameters of the patients. In principle, cameras will collect both 2D and 3D images to facilitate the extraction of events (eg, falls, loss of balance, wandering episodes), point trajectories, and “skeleton” data from patients for extraction of different motor behaviors and movements (eg, freezing, festination). The selected camera is the Intel RealSense depth camera (model D435)
- Tablet or computer to collect data related to cognitive abilities of the patients: the cognitive games app includes 6 different games about short-term memory, visual recognition, understanding, semantic memory, vocabulary, and mathematics. This app collects not only the results of the games, but also information related to the user interaction with it
- A local computer collects data from the patients via the sensorial ecosystem, which includes several fixed and dynamic sensors such as wearables, sensors, and cameras

In addition, the PC4L integrated care platform (accessible via internet browsers) provides an electronic health record or personal information sheet (fed with data provided by the PC4L ecosystem) and complex interface for personalized interaction and communication between patients and health care professionals.

#### PC4L Mobile App

The PC4L app includes a prescription dashboard, which monitors drug compliance; questionnaires on health status and physical activity, to be filled periodically; a symptom diary, which registers the symptoms experienced by the patient (ie, falls, freezing, loss of balance); physical activity recommendations; and daily personalized notifications on physical activity, hydration, and nutrition.

#### Decision Support System

As part of the decision support system, the PC4L platform includes a recommendation component. This component collects information from different sources available (directly from sensors, from the cognitive games, from the patients’ and users’ questionnaires, and from the multimodal fusion engine), and under clinical and health care professional guidance, after the assessment of the potential improvement or worsening of the patients’ conditions, issues personalized recommendations to address the problems identified. Recommendations are related to the following major areas: physical activity, sleep, cognitive, nutrition, social support, and stress management (for informal caregivers).

These recommendations are sent generally to the PC4L app installed on the patients’ mobile, where they appear as pop-up notifications when received. In most cases, the content of the notifications is readily available in the app in case the patient wants to check it again at a later time.

Under clinical supervision, the decision support system will indicate scores’ impacts and the contributions to either the improvement or the worsening of the patients’ health conditions. Finally, these decisions lead the recommender engine to provide personalized recommendations such as physical activity, nutrition, social support, and stress management. Additionally, reminders for therapy adherence to improve patients’ quality of life can be integrated.

Multimodal fusion aims to predict deviations from patients’ daily/normal routines, taking into consideration the input modalities (ie, cognitive state, medication intake, motor symptoms, sleep, physical activities, comorbidities, and patient’s profile). It focuses on detecting deviations in motor functions, cognitive abilities, sleep routines, and physical activities. The fusion approach benefits from domain knowledge and uses it in a computational framework to fuse the input modalities and detect the probability of deviations in the patient’s condition to support them. The multimodal fusion brings together the input modalities to enhance decision-making and gives stronger inferences using dynamic and personalized fusion through Bayesian networks. In the fusion approach, health care professionals use the patients’ profile information and the static scores to incorporate their knowledge regarding a patient into the fusion algorithm. In other words, the multimodal fusion algorithm utilizes the long-term analysis of the behavioral analysis modalities to gather broader insights into the deviations in patients’ health conditions and routines.

#### Metrics

During the monitoring period, specific metrics (Table 1) are collected, continuously and passively, through the PC4L system.

**Table 1:** Metrics and related devices used during patients’ monitoring.

Metrics	Devices
Walking parameters	Wristband and phone
Bradykinesia	Wristband and phone
Loss of balance	Cameras
Falls	Cameras and wristband
Festination	Wristband and phone and camera
Physical activity	Wristband
Freezing	Wristband and phone and camera
Inactivity	Wristband
Compliance with medication	App
Sleeping pattern	Wristband
Interaction patterns with the devices	Platform or app
Usage of the physical activity recommendation services	Number of clicks per week of the different services

#### PC4L System Versions

**Figure 1:**
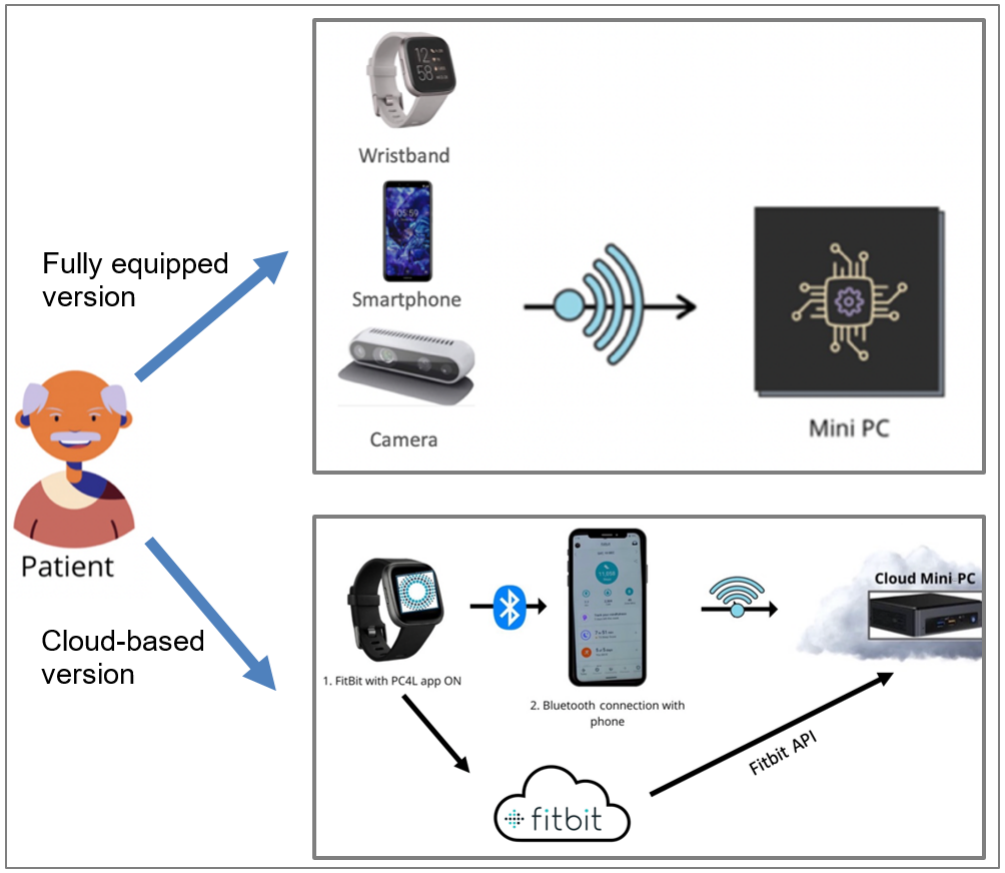
Illustrations of the PC4L solutions. API: application programming interface; PC4L: Personalized Integrated Care Promoting Quality of Life for Older People.

Two different versions of the PC4L system were used (Figure 1): a fully equipped version, with all the devices enumerated above provided to the patients, and a cloud-based system that only requires a smartphone and a wearable sensor. Of those patients using the PC4L system, 25.1% (70/279) used the fully equipped version, whereas the remaining ones (209/279, 74.9%) used the cloud-based system.

### Study Design

A prospective multicenter clinical study is conducted in 5 European countries: Germany, Italy, Portugal, Romania, and Spain (also including 6 pilot centers).

The pilot sites are (1) Asociacion Parkinson Madrid (APM), Madrid (Spain); (2) Casa di Cura del Policlinico (CCP), Milano (Italy); (3) Campus Neurológico Sénior (CNS), Lisbon (Portugal); (4) University and Emergency Hospital of Bucharest (UHB), Bucharest (Romania); (5) University of Medicine and Pharmacy (UMF), Bucharest (Romania); and (6) Wohlfahrtswerk für Baden-Württemberg (WBW), Stuttgart (Germany).

There are also 3 different scenarios: (1) rehabilitation center (inpatients and outpatients); (2) day care centers; and (3) home set (ie, patients’ home).

The duration per patient, at home and in the day care center, will be up to 3 months. Participants in the rehabilitation center participated during the period of their stay in the center (not exceeding the 3 months).

**Figure 2:**
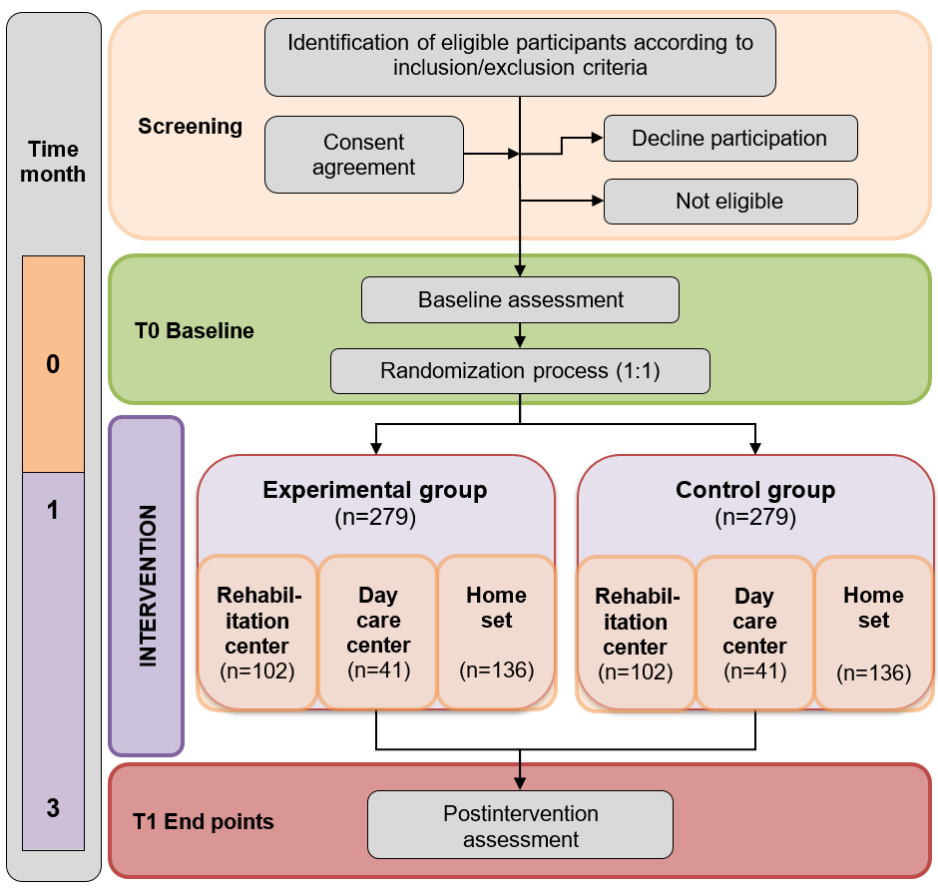
Flowchart of the proposed randomized controlled trial according to the CONSORT (Consolidated Standards of Reporting Trials) guidelines.

**Figure 3:**
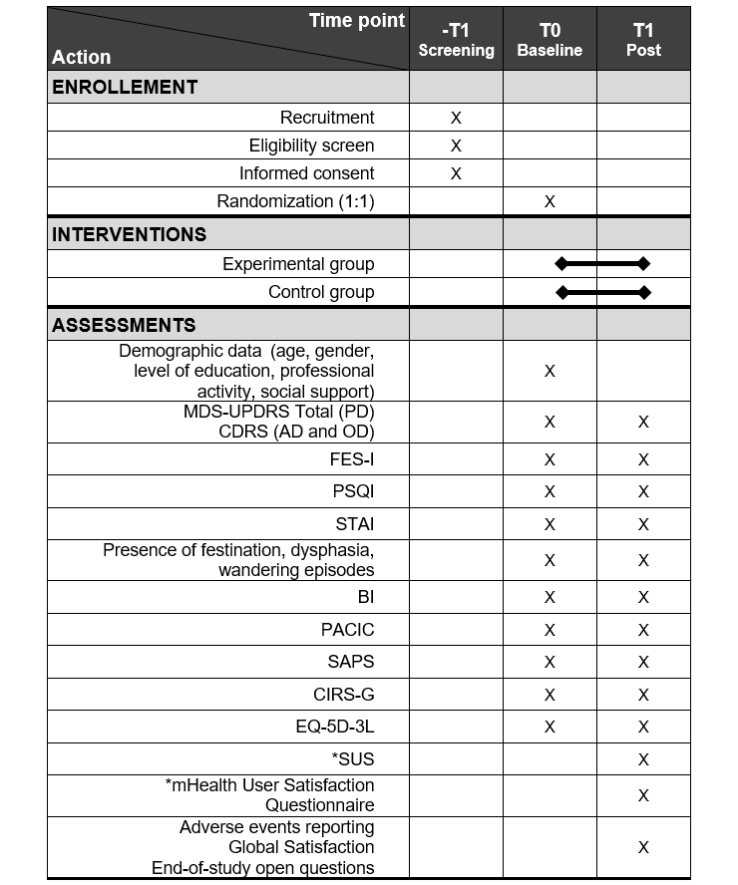
SPIRIT (Standard Protocol Items: Recommendations for Interventional Trials) diagram describing the schedule of enrollment, interventions, and assessments. *only for the EG intervention branch. AD: Alzheimer disease; BI: Barthel Index; CDRS: Clinical Dementia Rating Scale; CIRS-G: Cumulative Illness Rating Scale-Geriatric; FES-I: Falls Efficacy Scale International; MDS-UPDRS: Movement Disorder Society -Unified Parkinson's Disease Rating Scale; mHealth: mobile health; OD: other dementia; PACIC: Patient Assessment of Chronic Illness Care electromyography; PD: Parkinson disease; PSQI: Pittsburgh Sleep Quality Index; SAPS: Short Assessment of Patient Satisfaction; STAI: State-Trait-Anxiety-Inventory; SUS: System Usability Scale.

A specific route diagram is presented in Figure 2 in accordance with the CONSORT (Consolidated Standards of Reporting Trials) guidelines. The protocol of this study was developed in accordance with the SPIRIT (Standard Protocol Items: Recommendations for Interventional Trials) guidelines (Figure 3) [12]. The schedule of enrollment, interventions, and assessments is presented in Figure 3.

### Selection Criteria

The inclusion and exclusion criteria of participants are shown in Textbox 1.

**Participant inclusion and exclusion criteria.**

**Inclusion criteria**

- For patients with Parkinson disease (PD) and Parkinsonism:
  Patients diagnosed with PD according to the Movement Disorder Society (MDS) criteria
  Hoehn and Yahr stages between II and IV assessed in the “on” state
- For patients with Alzheimer disease (AD) or other dementia (OD):
  Clinical diagnosis of AD or OD
  General inclusion criteria
  Willing to participate
  Aged 65 years or older
  Willing to interact with technology devices
  Willing and able to provide written informed consent to participate in the study or having a legal representative responsible for signing

**General exclusion criteria**

- Clinically significant and severe cognitive decline or intellectual disability that may compromise their ability to use a mobile phone or other electronic devices
- Fever or acute infection such as COVID-19 or influenza
- Life-threatening coexisting disease with life expectancy

**Additional criteria regarding the different scenarios**

- Home set scenario: Living at home with or without caregiver
- Rehabilitation center scenario: Enrolled in a rehabilitation program with or without caregiver
- Day care center scenario: Being in the day care center program with a caregiver

### Recruitment

Patients with PD and other parkinsonian syndromes, AD, or ODs were invited to participate from the clinical centers involved in the study. Each participant organization was assigned to recruit a number of participants related to their skills, experience, number of researchers involved, and access to users (Table 2).

**Table 2:** Distribution of participants per clinical center and scenario.

Scenario/clinical center	Rehabilitation (n=204), n	Day care (n=82), n	Home (n=272), n	Total (N=558), n
APM^a^	80	N/A^b^	26	106
CCP^c^	63	N/A	62	125
CNS^d^	46	N/A	86	132
UHB^e^	15	N/A	15	30
UMF^f^	N/A	30	30	60
WBW^g^	N/A	52	53	105

^a^APM: Asociacion Parkinson Madrid.

^b^N/A: not applicable.

^c^CCP: Casa di Cura del Policlinico.

^d^CNS: Campus Neurológico Sénior.

^e^UHB: University and Emergency Hospital of Bucharest.

^f^UMF: University of Medicine and Pharmacy.

^g^WBW: Wohlfahrtswerk für Baden-Württemberg.

To reach the planned number of recruits, many strategies were implemented: participants were contacted through online webinars; through brochures placed in specific centers such as pharmacies, leisure centers, and medical offices; and through patients’ associations. Further, regular updates were posted via online newsletters on different websites, such as the PC4L website and end users organizations’ websites. In addition, information about the PC4L pilot 3 study was shared during national and international conferences. Recruitment was also promoted in a collaborative way thanks to partners from other European projects. Finally, participants were recruited in person when accessing the medical facilities and via phone contact or emails after searching local databases.

Patients who discontinue the study participation because of medical conditions (ie, infections, falls, medical emergencies) or unwilling to participate any longer for other reasons (ie, difficulty with technology, loss of interest) will be considered dropouts.

### Randomization

Patients were randomized in a ratio of 1:1 to the intervention group (use the PC4L system) or the control group (no intervention) by an independent, nonclinical partner of the project consortium.

Patients allocated in the intervention group were randomized in a ratio of 3:1 to the “cloud-based solution” and the “fully equipped” PC4L system version.

### Blinding

Participants and intervention delivery facilitators could not be blinded to group allocation. Evaluators conducting the clinical assessments were blinded to group allocation.

### Interventions

#### Overview

All participants that fulfil inclusion criteria were invited to participate. Information about objectives, duration, procedures, and voluntariness was given to patients and informed consent was obtained. None of the procedures of the study started before the informed consent had been signed by the participants.

#### Control Group

The patients allocated in the control group, after the baseline assessment, received general written recommendations on the aforementioned major areas (physical activities, sleep, cognitive, and nutrition; social support and stress management for caregivers). At the end of the study period, patients performed end-of-study evaluations.

#### Intervention Group

The patients recruited and assigned to the experimental group received specific instruction in using the project platform, but without any specific program concerning the activities.

Specifically, patients, after the baseline assessment, received the instrumental setup and indications on its functionalities. Patients were instructed about the use of the Fitbit, the PC4L app, and the cognitive games on a tablet or computer. Patients were also provided with manuals.

Specifically, according to the study approach detailed by Biehl et al [9], the patients were instructed to complete the following questionnaires in the app during the first week and during the last week of the study period: the 10-item Eating Assessment Tool (EAT10) [13] for evaluation of dysphagia; the Edmonton Symptom Assessment System (ESAS) [14] and the Patient Global Impression (PGI)—Severity [15] for evaluation of symptoms; and the International Physical Activity Questionnaire (IPAQ) [16] for evaluation of physical activity. In addition, during the last week of using the app, patients were requested to complete the SUS [17], via the app, for evaluation of usability.

The platform was installed in each scenario with the assistance of the project research staff (ie, bioengineers).

A total of 2 versions of PC4L systems were installed: while the cloud-based system (which only required a smartphone and a wearable sensor) was used in all scenarios, a fully equipped version was implemented only in the rehabilitation scenario.

The patients received the same directions in all 3 scenarios.

At the end of the study period, patients performed end-of-study evaluations and returned the instrumental setup.

### Study Assessments

In-person assessments took place at baseline and at the end of the study (3 months follow-up or at discharge from the rehabilitation program).

Specifically, the sample characterization and clinical evaluation of the participants (both experimental and control group patients) were performed at baseline. The clinical evaluation with specific tests and scales was repeated at the end of the study.

At the beginning and at the end of the study, all patients were assessed on quality of life through a specific scale (EQ-5D-3L).

Only for patients assigned to the intervention group, their experience with the PC4L platform was assessed in terms of usability at the end of the experimental period.

Figure 3 and the “Data Collection and Analysis” section report specific outcomes that will be used.

### Data Collection and Analysis

#### Clinical Assessment

Evaluators (ie, medical doctor, physical therapist, and neuropsychologist), at the beginning and at the end of the study, conducted the following clinical evaluation scales and tests:

- The Movement Disorder Society-Unified Parkinson Disease Rating Scale (MDS-UPDRS) [18] will be used to objectively quantify the motor and nonmotor aspects of PD. Changes in the MDS-UPDRS score will be investigated, with higher scores meaning worse outcome
- The Clinical Dementia Rating Scale (CDRS) [19] will be used to assess the cognitive status of AD and OD. It yields a single score on a scale of 0-5, with higher scores indicating worse outcome. Change in the CDRS score will be measured at baseline and at the end of the study
- The FES-I will be used to evaluate the subjective fear of falling of the patients. It yields a single score on a scale of 16-64, with higher scores indicating worse outcome
- The Pittsburgh Sleep Quality Index (PSQI) [20] will be used to evaluate the quality of sleep. Change in the PSQI score will be measured at baseline and at the end of the study. It yields a single score on a scale of 0-21, where higher scores mean worse outcome
- The State-Trait-Anxiety-Inventory (STAI) [21] will be used to assess the anxiety status (trait and state). Change in the STAI score will be measured at baseline and at the end of the study. It yields a single score on a scale of 20-80, where higher scores mean worse outcome
- Presence of festination, dysphasia, wandering episodes, and nonprogrammed medical resources use in the last month will be assessed (eg, access to emergency room)
- The Barthel Index (BI) is an ordinal scale used to measure performance in activities of daily living (ADLs) [22]. Each performance item is rated with a given number of points assigned to each level or ranking. It uses 10 variables describing ADL and mobility and yields a single score on a scale of 0-100, where higher scores mean better outcome
- The PACIC will be used to determine the involvement of the patient in his/her care plan. Change in the PACIC score will be measured at baseline and at the end of the study. It yields a single score on a scale of 0-5, where higher scores mean better outcome
- Similarly, the SAPS was used to evaluate the user’s satisfaction with respect to the medical treatment received. Change in the SAPS score will be measured at baseline and at the end of the study. It yields a single score on a scale of 0-28, where higher scores mean better outcome
- The Cumulative Illness Rating Scale-Geriatric (CIRS-G) [23] is used to include the presence of comorbidities in the clinical profile. Change in the CIRS-G score will be measured at baseline and at the end of the study. It yields a single score on a scale of 0-56, where higher scores mean worse outcome

In addition, patients’ characterization in terms of demographic and clinical data (age, gender, level of education, professional activity, social support, and previous use of technology) was performed.

Demographic and clinical data were aggregated, extracted, and analyzed using dedicated spreadsheet files (Microsoft Corporation).

#### Quality of Life Assessment

The primary outcome of the study will be the difference in quality of life between groups, measured by the EQ-5D-3L scale [24], before and after the experimentation.

The EQ-5D-3L descriptive system comprises 5 dimensions, namely, mobility, self-care, usual activities, pain/discomfort, and anxiety/depression. Each domain was scored by patients through 3 levels: no problems (1), some problems (2), and extreme problems (3) in the execution of the specific item.

Quality of life data were analyzed using dedicated spreadsheet files.

#### Usability and Satisfaction Assessment

At the end of the experimental period, patients allocated to the experimental group performed the usability evaluation of the PC4L platform solution by means of the SUS [17], Global Satisfaction and mHealth User Satisfaction questionnaires, adverse events reporting, and end-of-study open questions.

The SUS is used to measure how easy or difficult the proposed system is to use. We will compare the usability of 2 different versions of the PC4L system (fully equipped and cloud-based solutions). The overall SUS score will be calculated and assessed consistently with the literature: a score of 100 corresponds to the best imaginable usability, while acceptable usability is defined by an SUS score above 68 points [17,25].

The Global Satisfaction Questionnaire is used to assess the overall level of satisfaction or dissatisfaction with the platform experience.

The mHealth User Satisfaction questionnaire is used to measure user satisfaction and usability of the recommendation component of the solution [26].

As a completion of end-of-study assessment, an adverse events report and end-of-study open questions of preferred and less preferred aspects of the platform, as well as suggestions for improvements, will be collected for each user.

### Sample Size

According to published evidence [27], the minimal clinically important difference using the EQ-5D-3L in patients with PD is 5.23 points. The sample size calculation was based on these values and on the data provided in a previous study [28] in the PD field that compared the quality of life of 2 groups of patients, resulting in 10% of difference between groups, corresponding to 6 points. It was estimated that 558 patients, 279 per study group, are necessary to improve 10% in the EQ-5D-3L score, with 95% power. Dropouts were not considered in the calculation. Based on this result, the number of participants was distributed among clinical centers, according to their recruitment capacity (Table 2).

### Statistical Analysis

Descriptive analysis will be performed for all variables to characterize the sample. For continuous variables, mean, SD, median, and range values will be calculated. For categorical variables, absolute and relative frequencies will be used to describe the data [29-31].

Normality will be studied using the Kolmogorov-Smirnov and Shapiro-Wilk tests. The effect of using the PC4L system will be analyzed by applying the paired-sample *t* test (2-tailed) and the Wilcoxon signed rank test to each variable (statistical significance will be set at P<.05).

The Pearson and Spearman correlation tests will be used to validate the PC4L systems metrics (statistical significance will be set at P<.05).

### Data Collection and Management

The information will be handled confidentially to prevent participant names or other directly identifiable information from appearing in any report, publication, or other disclosure of results. Data (eg, name, age, length of illness) are collected and stored in separate folders. The investigator will enter the pseudonymous data into a database for statistical analysis.

Essential documents shall be archived in such a way as to ensure that they are readily available, on request, to the competent authorities.

The collected data of the PC4L components are only made available in a pseudonymized form in the secure cloud “Amazon Web Services,” which is located within Europe [32]. The patient will therefore be assigned a code to ensure the pseudonymity of his/her data. The data on the cloud are only accessible to the project partners, with whom a data processing agreement was made.

The data processing agreement was stipulated as an agreement between the data controller (partner 1, ie, clinical partners collecting the data) and a data processor (partner 2, ie, partner responsible for data analysis) within the consortium, with the purpose of regulating the processing of any personal data collected. In the context of joint controllers, partner 1 is responsible for the processing of personal data in a conventional form from participants (health data and medical data set derived from patients involved in the user requirements phase and in the pilots, data from caregivers, professionals, and other stakeholders). Partner 2 is—within the framework of joint controllers—responsible for the processing of personal data in device data and annotations (social and medical data set/audiovisual).

The data obtained will be used only for the purposes of the research and not for other purposes. The results of the research will be shared more widely, for example, through publications and conferences, but no personal information will be displayed. The processing, communication, and transfer of personal data will be carried out in accordance with the provisions of Regulation (EU) 2016/679 [33] and Law 3/2018 [34], of 5 December, on the Protection of Personal Data and the Guarantee of Digital Rights. In accordance with the legislation, the participant may exercise his/her rights of cancellation, opposition, portability, limitation, access, and rectification by contacting the professional who informed the participant about the project. Likewise, the consortium adheres to the principles of the General Data Protection Regulation (GDPR; legality, equity and transparency; purpose limitation; minimization of data; accuracy; storage limitation; integrity and confidentiality; and responsibility).

Any inaccuracy regarding the functioning of the system will be promptly reported to the technical partners of the project who will intervene in correcting them. The interruption of operation of 1 or more components of the PC4L system will not have any kind of feedback/effect on the participant.

### Ethics Approvals

#### Study Approval

The study protocol was reviewed and approved by the local ethical committees in Germany [number 2022-29-MB-FA4, Ethical committee of Westfälische Wilhelms-Universität (WWU), Münster], Italy (number 734-2022bis, Comitato Etico Milano Area 2), Portugal (number 6-2022 R, CNS Ethic Committee), Romania (numbers 7/19.07.2022, Comisia de Etică a Cercetării din Spitalul Colentina and 36432, Comitetul local de etică SUUB/ Spitalul Universitar de Urgență București), and Spain (number 22/392-E, Ethical committee of Hospital Clinico San Carlos – Salud Madrid). The institutional review board of all centers approved the study and will receive study reports during mid-study and at the end of the study, and will monitor the study implementation and data collection. Any deviations from the protocol will be promptly notified to the ethics committees and applied only after its approval.

The randomized controlled trial has been registered at ClinicalTrials.gov (identifier NCT05538455).

The study is conducted in accordance with the principles of the Declaration of Helsinki.

The results of this study will be disseminated through peer-reviewed journals and national and international academic conferences only by the professionals directly involved in the clinical trial.

#### Patient’s Informed Consent

After the study had been fully explained to the patient, written informed consent was obtained prior to any study-related proceedings. The method of obtaining and documenting the informed consent and the contents of the consent comply with ICH-GCP (International Conference on Harmonisation good clinical practice), all applicable regulatory requirements, and legal requirements.

In case the patient is unable to read or sign, a legal guardian is allowed to sign the informed consent, only after confirming that the study has been comprehensively explained to the patient by the research team.

#### Security and Adverse Event Reporting

Any adverse event (serious or nonserious) and any other abnormal clinical, personal, social finding will be recorded in the patient’s case report form. For all adverse events, sufficient information will be pursued to permit the following:

- a determination of a temporal relationship;
- an adequate determination of the outcome of the event (ie, whether the event should be classified as a serious adverse event); and
- an assessment of the casual relationship between the adverse event and the study requirements.

An abnormal result finding will be classified as an adverse event if 1 or more of the following criteria are met:

- the result finding leads to a discontinuation of patient participation in the research study and
- the result finding is considered an adverse event by the researcher.

### Withdrawal of Participants

Participation in this study is voluntary. Patients may withdraw from the study at any time, without giving reasons and without disadvantage in terms of the quality of care they would receive if they did not participate. After the withdrawal, no further data will be collected or taken into consideration for statistical purposes.

## Results

The recruitment began in September 2022, and enrollment ended in February 2023. Recruitment is now closed (April 2023). The results of this study are expected to be published during the summer 2023.

The results of this study will be presented at national and international patients’ organizations and to the public.

The study protocol is part of a project (grant agreement number 875221) approved and funded by the European Commission in the framework of Horizon 2020 action.

The results of pilot 3 will allow clarification of the usability of the final version of the PC4L system, its impact on patients’ and caregivers’ quality of life, empowerment, and use of medical resources. It would also provide information on the support of the system as a tool to facilitate the decision-making process.

At the time of the deployment of this study, as for the previous protocols, issues due to the COVID-19 pandemic in European countries were managed to avoid safety-related risks.

## Discussion

PC4L recognizes that today in health care an integrated care process should be adopted for harmonizing health models with social services from a holistic perspective [35]. Therefore, the project intends to develop and test a technology-based, integrated, scalable, and interactive care platform by involving patients with neurodegenerative diseases, such as PD and dementias. The principal aims of this project are to improve the quality of life of patients and to enable active living and better disease management. These aims will be achieved through large-scale assessments across Europe (in 5 countries and 6 end user organizations), with the final scope to validate the reliability of the overall system in a real-life context.

Eventually, the main advantage of the PC4L project is to propose a system that can be easily adapted to the reality of various chronic diseases, care institutions, and end user needs, thus benefiting all actors involved.

To assess the impact on quality of life and usability, we decided to adopt a randomized controlled trial model because it minimizes selection bias and allows reaching an adequate sample size to achieve power.

In the context of improved technologies and digitalization strategies, the use of wearable sensors and smartphone apps has the advantage of providing objective, longitudinal, and precise information about the status of the patients in their own home environment, as highlighted by Klucken et al [36], especially in cases of neurodegenerative diseases.

Based on the extensive literature on the benefit of physical activity in neurodegenerative diseases [37-46], we expect an improvement in quality of life. This is also facilitated by the early detection of signs and symptoms (eg, falls) through proactive monitoring.

Moreover, in terms of digital health, the inclusion of participants is a crucial part [47]. Based on our recent work, we considered that the ICT-integrated care platform should not promise functionalities that are too complex for too many users [11]. Instead, we focused on selection and restriction to fewer but essential aspects. Users and other stakeholders have in fact been involved in these decisions by being considered active actors from the early stages of the project. Notably, the clinical study here described is conducted in 3 different real-life scenarios, providing relevant information on the everyday life of the patients.

Regarding the home scenario, the primary advantage of implementing this scenario is the ability to monitor patients at home, measuring their general health condition, asking about their perceived state, and detecting their symptoms (eg, gait disturbances or falls). The acquisition of these data will enrich patients’ perception of their own condition and will allow the comparison between their insight and the clinical and instrumental data. Health professionals, from their perspective, can take advantage of all these data by adapting the prescribed therapy in a highly personalized manner. Thus, patient-doctor communication will be improved, facilitating patients’ engagement with their own health care process.

Central to the study is also the implementation of the PC4L solution in the rehabilitation centers, as such controlled environments involve patients in different kinds of activities, under the supervision of a multidisciplinary team. Here, patients perform personalized treatments in dedicated rooms, such as motor training and exercises connected to ADL, as well as cognitive exercises to improve memory and attention. The main advantages of PC4L in this scenario are (1) the possibility to provide objective information to professionals to study the evolution, treatment personalization, and rehabilitation suggestions; (2) to check the communication between professionals, patients, and caregivers, especially exercise recommendations based on the evolution of patient’s movement skills; and (3) to design a decision support tool taking into account the patient’s personal and health condition.

Finally, in the third scenario of day care centers, patients perform activities connected to ADL like at home, but under the supervision of professionals (day care center workers). Additionally, the participation of caregivers in this scenario can support the patients. The benefits are to check the communication between professionals and the caregiver, who does not live in the same house with the patient, and to monitor the patient’s evolution.

In this pilot study, as already reported by Espay et al [48], low digital skills could be a limitation to the appropriate use of the PC4L solution and, therefore, the correct monitoring of the participants. The consortium mitigated this risk by adopting simplified age-appropriate questionnaires and tools.

Another risk could be the completion of the recruitment of the patients: for this purpose, the consortium of PC4L has adopted several mitigation measures (described earlier).

Trying to provide a balance between an acceptable benefit-to-burden ratio and the delivery of reliable, clinically relevant insights, through mobile health (mHealth) technologies, as suggested by Espay et al [49], the main solution of PC4L includes the continuous use of the wristband for monitoring parameters such as steps and levels of physical activity, and the PC4L app, which includes easy but engaging functionalities.

The final goal of the PC4L project is to obtain a good coordinated care model between all involved actors. Future developments of such projects might dwell into the active involvement of caregivers and socio-health professionals and into the support of professionals in the decision-making process, to facilitate efficient communication between all stakeholders and ensure reliable and protected access to data within Europe [8].

## Abbreviations

AD: Alzheimer disease
ADL: activity of daily living
APM: Asociacion Parkinson Madrid
BI: Barthel Index
CCP: Casa di Cura del Policlinico
CDRS: Clinical Dementia Rating Scale
CIRS-G: Cumulative Illness Rating Scale-Geriatric
CNS: Campus Neurológico Sénior
CONSORT: Consolidated Standards of Reporting Trials
EAT10: 10-item Eating Assessment Tool
ESAS: Edmonton Symptom Assessment System
FES-I: Falls Efficacy Scale-International
GDPR: General Data Protection Regulation
ICH-GCP: International Conference on Harmonisation good clinical practice
IPAQ: International Physical Activity Questionnaire
MDS: Movement Disorder Society
MDS-UPDRS: Movement Disorder Society-Unified Parkinson Disease Rating Scale
OD: other dementia
PACIC: Patient Assessment of Chronic Illness Care
PC4L: Personalized Integrated Care Promoting Quality of Life for Older People
PD: Parkinson disease
PGI: Patient Global Impression
PSQI: Pittsburgh Sleep Quality Index
SAPS: Short Assessment of Patient Satisfaction
SPIRIT: Standard Protocol Items: Recommendations for Interventional Trials
STAI: State-Trait-Anxiety-Inventory
SUS: System Usability Scale
UHB: University and Emergency Hospital of Bucharest
UMF: University of Medicine and Pharmacy
WBW: Wohlfahrtswerk für Baden-Württemberg

## Data Availability

The data sets generated during or analyzed during this study will be available from the corresponding author on reasonable request.

## References

[1] Guthmuller S, Paruolo P, Verzillo S, Baltagi BH, Moscone F (2021). Positive externalities of EU actions on sustainability of health systems. The Sustainability of Health Care Systems in Europe.

[2] GBD 2016 Parkinson's Disease Collaborators (2018). Global, regional, and national burden of Parkinson's disease, 1990-2016: a systematic analysis for the Global Burden of Disease Study 2016. Lancet Neurol.

[3] Ferri CP, Prince M, Brayne C, Brodaty H, Fratiglioni L, Ganguli M, Hall K, Hasegawa K, Hendrie H, Huang Y, Jorm A, Mathers C, Menezes PR, Rimmer E, Scazufca M (2005). Global prevalence of dementia: a Delphi consensus study. The Lancet.

[4] Broese van Groenou MI, De Boer A (2016). Providing informal care in a changing society. Eur J Ageing.

[5] Odone A, Buttigieg S, Ricciardi W, Azzopardi-Muscat N, Staines A (2019). Public health digitalization in Europe. Eur J Public Health.

[6] Block VAJ, Pitsch E, Tahir P, Cree BAC, Allen DD, Gelfand JM (2016). Remote Physical Activity Monitoring in Neurological Disease: A Systematic Review. PLoS One.

[7] Lorenz K, Freddolino PP, Comas-Herrera A, Knapp M, Damant J (2019). Technology-based tools and services for people with dementia and carers: mapping technology onto the dementia care pathway. Dementia (London).

[8] van Halteren AD, Munneke M, Smit E, Thomas S, Bloem BR, Darweesh SKL (2020). Personalized care management for persons with Parkinson’s disease. J Parkinson's Dis.

[9] Biehl V, Becker H, Ogrin A, Reissner A, Burger J, Glaessel A (2021). User-centered development of a web platform supporting community-based health care organizations for older persons in need of support: qualitative focus group study. J Med Internet Res.

[10] Ahmed M, Marín M, Bouça-Machado R, How D, Judica E, Tropea P, Bentlage E, Brach M (2021). Investigating users' and other stakeholders' needs in the development of a personalized integrated care platform (PROCare4Life) for older people with dementia or Parkinson disease: protocol for a mixed methods study. JMIR Res Protoc.

[11] Ahmed M, Marín M, How D, Judica E, Tropea P, Bentlage E, J Ferreira J, Bouça-Machado R, Brach M (2022). End users' and other stakeholders' needs and requirements in the development of a personalized integrated care platform (PROCare4Life) for older people with dementia or Parkinson disease: mixed methods study. JMIR Form Res.

[12] Chan A, Tetzlaff J, Gøtzsche PC, Altman D, Mann H, Berlin J, Dickersin K, Hróbjartsson A, Schulz KF, Parulekar WR, Krleza-Jeric K, Laupacis A, Moher D (2013). SPIRIT 2013 explanation and elaboration: guidance for protocols of clinical trials. BMJ.

[13] Belafsky PC, Mouadeb DA, Rees CJ, Pryor JC, Postma GN, Allen J, Leonard RJ (2008). Validity and reliability of the Eating Assessment Tool (EAT-10). Ann Otol Rhinol Laryngol.

[14] Hui D, Bruera E (2017). The Edmonton Symptom Assessment System 25 Years Later: Past, Present, and Future Developments. J Pain Symptom Manage.

[15] Srikrishna S, Robinson D, Cardozo L (2010). Validation of the Patient Global Impression of Improvement (PGI-I) for urogenital prolapse. Int Urogynecol J.

[16] Lee PH, Macfarlane DJ, Lam TH, Stewart SM (2011). Validity of the International Physical Activity Questionnaire Short Form (IPAQ-SF): a systematic review. Int J Behav Nutr Phys Act.

[17] Brooke J, Jordan PW, Thomas B, Weerdmeester BA, McClelland IL (1996). SUS -- a quick and dirty usability scale. Usability Evaluation in Industry.

[18] Movement Disorder Society Task Force on Rating Scales for Parkinson's Disease (2003). The Unified Parkinson's Disease Rating Scale (UPDRS): status and recommendations. Mov Disord.

[19] Tan JE, Strauss E, Sherman EMS, Kreutzer JS, DeLuca J, Caplan B (2011). Clinical Dementia Rating. Encyclopedia of Clinical Neuropsychology.

[20] Buysse DJ, Reynolds CF, Monk TH, Berman SR, Kupfer DJ (1989). The Pittsburgh Sleep Quality Index: a new instrument for psychiatric practice and research. Psychiatry Res.

[21] Marteau TM, Bekker H (1992). The development of a six-item short-form of the state scale of the Spielberger State-Trait Anxiety Inventory (STAI). Br J Clin Psychol.

[22] Shah S, Vanclay F, Cooper B (1989). Improving the sensitivity of the Barthel Index for stroke rehabilitation. J Clin Epidemiol.

[23] Linn B S, Linn M W, Gurel L (1968). Cumulative illness rating scale. J Am Geriatr Soc.

[24] Brooks R, Rabin R, De Charro F (2013). The measurement and valuation of health status using EQ-5D: a European perspective: evidence from the EuroQol BIOMED Research Programme.

[25] Bangor A, Kortum P, Miller J (2009). Determining what individual SUS scores mean: adding an adjective rating scale. Journal of Usability Studies.

[26] Melin J, Bonn SE, Pendrill L, Trolle Lagerros Y (2020). A questionnaire for assessing user satisfaction with mobile health apps: development using Rasch Measurement Theory. JMIR Mhealth Uhealth.

[27] Winter Y, Lubbe D, Oertel W, Dodel R (2012). QL2 evaluation of minimal clinically important differences for health-related quality of life scales in Parkinson's disease. Value Health.

[28] Fan X, Wang D, Hellman B, Janssen M, Bakker G, Coghlan R, Hursey A, Matthews H, Whetstone I (2018). Assessment of health-related quality of life between people with Parkinson's disease and non-Parkinson's: using data drawn from the '100 for Parkinson's' smartphone-based prospective study. Int J Environ Res Public Health.

[29] Kawulich B (2004). Data analysis techniques in qualitative research. Journal of Research in Education.

[30] Rabiee F (2004). Focus-group interview and data analysis. Proc Nutr Soc.

[31] O'Connor H, Gibson N (2003). A step-by-step guide to qualitative data analysis. PimatisiwA Journal of Indigenous and Aboriginal Community Health.

[32] Privacy Notice. Amazon.

[33] (2016). Regulation (EU) 2016/679 of the European Parliament and of the council of 27 April 2016 on the protection of natural persons with regard to the processing of personal data and on the free movement of such data, and repealing Directive 95/46/EC (General Data Protection Regulation). EUR-Lex.

[34] (2019). Organic Law 3/2018, of December 5, protection of personal data and guarantee of digital rights. European Commission.

[35] Chirra M, Marsili L, Wattley L, Sokol LL, Keeling E, Maule S, Sobrero G, Artusi CA, Romagnolo A, Zibetti M, Lopiano L, Espay AJ, Obeidat AZ, Merola A (2019). Telemedicine in Neurological Disorders: Opportunities and Challenges. Telemed J E Health.

[36] Klucken J, Krüger R, Schmidt P, Bloem BR (2018). Management of Parkinson’s disease 20 years from now: towards digital health pathways. J Parkinson's Dis.

[37] Caspersen CJ, Powell KE, Christenson GM (1985). Physical activity, exercise, and physical fitness: definitions and distinctions for health-related research. Public Health Rep.

[38] Chastin SF, Baker K, Jones D, Burn D, Granat MH, Rochester L (2010). The pattern of habitual sedentary behavior is different in advanced Parkinson's disease. Mov Disord.

[39] Crizzle A, Newhouse I (2006). Is physical exercise beneficial for persons with Parkinson's disease?. Clin J Sport Med.

[40] da Silva PGC, Domingues DD, de Carvalho LA, Allodi S, Correa CL (2016). Neurotrophic factors in Parkinson's disease are regulated by exercise: evidence-based practice. J Neurol Sci.

[41] Hillman CH, Erickson KI, Kramer AF (2008). Be smart, exercise your heart: exercise effects on brain and cognition. Nat Rev Neurosci.

[42] Hou L, Chen W, Liu X, Qiao D, Zhou F (2017). Exercise-Induced Neuroprotection of the Nigrostriatal Dopamine System in Parkinson's Disease. Front Aging Neurosci.

[43] Keus SH, Bloem BR, Hendriks EJ, Bredero-Cohen AB, Munneke M, Practice Recommendations Development Group (2007). Evidence-based analysis of physical therapy in Parkinson's disease with recommendations for practice and research. Mov Disord.

[44] Kwakkel G, de Goede C, van Wegen E (2007). Impact of physical therapy for Parkinson's disease: A critical review of the literature. Parkinsonism & Related Disorders.

[45] Lauzé Martine, Daneault Jean-Francois, Duval Christian (2016). The Effects of Physical Activity in Parkinson's Disease: A Review. J Parkinsons Dis.

[46] Petzinger GM, Fisher BE, McEwen S, Beeler JA, Walsh JP, Jakowec MW (2013). Exercise-enhanced neuroplasticity targeting motor and cognitive circuitry in Parkinson's disease. Lancet Neurol.

[47] Conard S (2019). Best practices in digital health literacy. Int J Cardiol.

[48] Espay AJ, Bonato P, Nahab FB, Maetzler W, Dean JM, Klucken J, Eskofier BM, Merola A, Horak F, Lang AE, Reilmann R, Giuffrida J, Nieuwboer A, Horne M, Little MA, Litvan I, Simuni T, Dorsey ER, Burack MA, Kubota K, Kamondi A, Godinho C, Daneault J, Mitsi G, Krinke L, Hausdorff JM, Bloem BR, Papapetropoulos S, Movement Disorders Society Task Force on Technology (2016). Technology in Parkinson's disease: challenges and opportunities. Mov Disord.

[49] Espay AJ, Hausdorff JM, Sánchez-Ferro Á, Klucken J, Merola A, Bonato P, Paul SS, Horak FB, Vizcarra JA, Mestre TA, Reilmann R, Nieuwboer A, Dorsey ER, Rochester L, Bloem BR, Maetzler W, Movement Disorder Society Task Force on Technology (2019). A roadmap for implementation of patient-centered digital outcome measures in Parkinson's disease obtained using mobile health technologies. Mov Disord.

